# Allier théorie et pratique dans la lutte contre la pandémie du Sida

**DOI:** 10.11604/pamj.2014.19.88.5116

**Published:** 2014-09-26

**Authors:** Roger Zerbo

**Affiliations:** 1 Institut des Sciences des Sociétés, UFR-SH Université de Ouagadougou, Ouagadougou, Burkina Faso

**Keywords:** Sida, VIH, Burkina Faso, AIDS, HIV, Burkina Faso

## Abstract

Le premier cas d'infection par le VIH a été notifié en 1986 au Burkina Faso. L'ignorance qui entourait cette infection et l'absence de traitement curatif a amplifié ses conséquences sociales et économiques. La mise en œuvre des interventions communautaire est un enjeu majeur actuel qui recommande la compréhension des logiques sociales endogènes qui influencent les comportements individuels et collectifs. C'est en cela que l'implication des sciences sociales dans la lutte contre les maladies, en particulier le Sida constitue un enjeu, en termes de définition et d'identification de leur contribution. Dans notre propos, nous pouvons mettre en évidence trois niveaux de contribution des sciences sociales, notamment la sociologie, l'anthropologie, la psychologie et dans une certaine mesure le droit et l’économie, à la prévention du Sida et la prise en charge des personnes infectées par le VIH. Il faut noter que ces disciplines contribuent à la lutte contre le VIH d'une part, par des réflexions et des éléments d'analyses constructives, et d'autre part, l'aptitude des porteurs de ces disciplines est parfois sollicitée pour l'efficacité de la mise en œuvre des actions et l'organisation des systèmes de soins.

## Aux éditeurs du Journal Panafricain de Médecine

Dans les années 1980, l'infection par le virus d'Immuno-déficience humaine (VIH) a été marquée par une ignorance de la maladie et l'absence de traitement curatif qui ont entrainé d’énormes conséquences sociales et économiques. Des interventions communautaires ont été recommandées pour remémorer les traitements et réduire la stigmatisation. L'anthropologie contribue à la compréhension des problèmes contemporains auxquels les communautés sont confrontées dans la dynamique du changement social. C'est en cela que l'implication des sciences sociales dans la lutte contre les maladies, particulièrement le sida, constitue un enjeu, pour la définition des modalités de leurs contributions. Nous mettons en évidence trois niveaux de contribution de la sociologie, la socio-anthropologie, la psychologie et dans une certaine mesure le droit et l’économie, la bioéthique à la prévention du sida et la prise en charge des personnes infectées. Ces disciplines contribuent à la lutte contre le VIH d'une part, par des réflexions et des éléments d'analyses constructives, et d'autre part, l'aptitude des porteurs de ces disciplines est sollicitée pour l'efficacité et l'efficience des systèmes de soins.

Les anthropologues ont été mobilisées dans une perspective de recherche opérationnelle qui aide à la prise de décision en matière de politique publique. En effet, une réalité relève du domaine de politique publique, lorsqu'elle est prise en compte par des acteurs autres que les cibles ou les premières victimes. Ce fut le cas du VIH qui a mobilisé des malades, des activistes, des politiques, des religieux, des chercheurs en génétique et bioéthique et peut-être humanistes. L'anthropologie, jadis orientée vers la recherche fondamentale est désormais ouverte à la recherche-action pour contribuer la l’épanouissement des populations vulnérables du fait de la maladie et la précarité. De nombreux agents de santé aux contacts des anthropologues, de leur démarche et la sensibilité qu'ils développent vis-à-vis des maladies, ont pris l'option de s'initier à ces sciences en vue d'améliorer leurs relations thérapeutiques.

### Apport des sciences sociales par l'ethnographie

Cette contribution se décline en termes d'apports à l'analyse et la compréhension des représentations de la maladie, la compréhension des comportements des populations face à la maladie, les itinéraires thérapeutiques. On a compris les perceptions de la maladie pour déconstruire les discours stigmatisants les personnes atteintes du VIH [1]. Ce qui permet aux personnes vivant avec le VIH de sortir de la clandestinité et intégrer des associations de prise en charge. Des analyses socio-comportementales accompagnées de plaidoirie ont été orientées sur la nécessité d'opter pour la gratuité des soins au profit des personnes infectées par le VIH, afin de leur assurer une bonne qualité de vie et leur productivité [2]. Le rapport à la mort et les contraintes de vivre avec une maladie chronique et incurable a été analysé et rapproché du contexte du sida pour aider à l’élaboration des messages d’éducations sanitaires pour la prévention. L'objectif était de pouvoir tenir un discours public sur la maladie sans accentuer ou provoquer des séries de psychoses collectives et mettre en place des mécanismes de prise en charge psychosociale des personnes infectées et affectées par l'impact du VIH. Les manières de percevoir la maladie à travers les nosographies populaires, les procédés diagnostics, déterminent les itinéraires thérapeutiques.

### Apport des sciences sociales par l'anthropologie critique

Cette contribution s'est traduite par des analyses critiques des politiques publiques de lutte contre le VIH. Les travaux de Laurent Vidal [3] en Afrique de l'Ouest et ceux de Paul Farmer [4] en Haïti, ont mis en évidence le caractère très politique de la lutte contre le VIH, montrant que la lutte contre le VIH est une des problématiques des droits humains fondamentaux. Autant soigner tout le monde sans discrimination [5]. Aussi, des populations peuvent avoir de très bonnes connaissances en techniques de prévention du VIH, les modes de transmission, les manifestations, les comportements à risque, sans pour autant utiliser les moyens recommandés pour se prémunir. Dans ce sens, Alain Epelboin [6] fait remarquer que de nombreux auteurs ont fait le constat du décalage entre le comportement des gens face au risque du VIH et leur propos. Dans de nombreuses études de faisabilité et d’évaluation d'impact des programmes de lutte contre de VIH les compétences en matière d'approche sociologique ou anthropologiques sont associées. Pour une pathologie, même si des remèdes sont disponibles, l'attitude des populations face à celle-ci, son acceptation, l'observance et la volonté de prévention méritent attentions.

### Apport des sciences sociales par l'implication des acteurs

Cette contribution des sciences sociales à la lutte contre le VIH se traduit par la participation des porteurs de ces disciplines à l'accompagnement des acteurs de lutte contre la maladie dans la mise en œuvre de leurs activités. Sans être les praticiens de la santé, des anthropologues ont été amenés à collaborer étroitement dans l'organisation du processus thérapeutique. Les analyses ont montré la pertinence d'organiser la lutte contre la maladie selon les catégories sociales et de tenir compte des conditions socio-économiques personnes concernées [7]. Des psychologues et des anthropologues ont été sollicités en tant que personnes ressources et requière pour cela leur aptitude à organiser les relations entre les acteurs et les institutions.

## Conclusion

La problématique du VIH est considérée en sciences sociales au même titre que les projets de développement et les dynamiques du changement social. Les enjeux sociaux et sanitaires imposés par le VIH ont induit un changement important dans l'approche de la socio-anthropologie et la démarche d'implication a favorisé des changements de perspectives dans la mise en œuvre des politiques publique.
